# Association between the triglyceride-glucose index and impaired cardiovascular fitness in non-diabetic young population

**DOI:** 10.1186/s12933-023-02089-8

**Published:** 2024-01-20

**Authors:** Dachuan Guo, Zhenguo Wu, Fei Xue, Sha Chen, Xiangzhen Ran, Cheng Zhang, Jianmin Yang

**Affiliations:** National Key Laboratory for Innovation and Transformation of Luobing Theory; The Key Laboratory of Cardiovascular Remodeling and Function Research, Department of Cardiology, Chinese Ministry of Education, Chinese National Health Commission and Chinese Academy of Medical Sciences, Qilu Hospital of Shandong University, Jinan, China

**Keywords:** Triglyceride-glucose index, Cardiovascular fitness, Young, Non-diabetes

## Abstract

**Background:**

The triglyceride-glucose (TyG) index has been linked to the onset, progression, and prognosis of cardiovascular disease (CVD) in middle-aged and elderly individuals. Nevertheless, the relationship between the TyG index and impaired cardiovascular fitness (CVF) remains unexplored in non-diabetic young population.

**Methods:**

We used data from the National Health and Nutrition Examination Survey (NHANES) study (1999–2004) to conduct a cross-sectional study of 3364 participants who completed an examination of CVF. Impaired CVF was defined as low and moderate CVF levels determined by estimated maximal oxygen consumption (Vo2max), based on sex- and age-specific criteria. The TyG index was calculated by $Ln[TG(mg/dL)\times FPG(mg/dl)/2]$.

**Results:**

The age (median with interquartile range) of the study population was 28 (19–37) years, and the TyG index (median ± standard deviation) was 8.36 ± 0.52. A significant association between the TyG index and impaired CVF was found in multivariable logistical regression analysis (per 1-unit increase in the TyG index: OR, 1.46; 95% Cl 1.13–1.90). A dose‒response relationship between the TyG index and impaired CVF was presented by restricted cubic splines (RCS). A significant interaction (*p* = 0.027) between sex and the TyG index for impaired CVF was found in the population aged < 20 years.

**Conclusions:**

In non-diabetic young population, individuals with higher TyG index values are at an increased likelihood of encountering impaired CVF. Furthermore, sex may exert an impact on CVF, as males tend to be more susceptible to impaired CVF under comparable TyG index conditions.

**Supplementary Information:**

The online version contains supplementary material available at 10.1186/s12933-023-02089-8.

## Background

Ischemic heart disease and stroke are the first and second causes of death in people over 50 years old, respectively [1]. A report from the American Heart Association indicated that heart disease and stroke had claimed more lives than the sum of cancer and chronic lower respiratory disease in 2020, and the crude prevalence of cardiovascular disease (CVD) in 2020 had a 29.01% increase compared with 2010 [2].

Cardiorespiratory fitness (CRF), quantified as maximal oxygen consumption (Vo2max), represents the holistic capability to efficiently transfer oxygen from the atmosphere to the mitochondria during physical activity [3]. CRF is a powerful predictor of all-cause and cardiovascular mortality [4, 5]. According to recent scientific statements issued by the American Heart Association, it was both important and feasible to measure CRF in the clinical environment [3, 6–10].

The triglyceride-glucose (TyG) index has been proven to be associated with incidence of CVD, and participants with the highest TyG index faced roughly double risk of coronary heart disease compared to those with the lowest TyG index [11, 12]. The main target population of these studies were elderly individuals. Although one study indicated that the TyG index can predict adverse cardiovascular events in patients with premature coronary artery disease, the average age of the study population was 44.48 ± 6.30 years [13]. Meanwhile, the TyG index, as an indicator of insulin resistance, has a tight relationship with type 2 diabetes mellitus. The prevalence of CVD in type 2 diabetes was as high as 32.2%, which was much higher than that in the general population [14]. A prospective cohort study suggested that the TyG index is still a predictive marker for CVD and CHD risk in non-diabetic population [15]. Up to now, there have been too few studies on the TyG index in non-diabetic young population.

We therefore sought to determine the association between the TyG index and impaired CVF in a non-diabetic young population.

## Methods

### Study design and population

The data analyzed in this study were from the 1999 to 2004 National Health and Nutrition Examination Survey (NHANES) database. The NHANES is a continuous survey that selects a representative group of Americans through complex multistage probability sampling to assess the health and nutritional status of American adults and children. The NHANES study plan was conducted with approval by the Ethics Review Committee of the National Center for Health Statistics. All study participants provided written informed consent. For more details, please visit NHANES—NCHS Research Ethics Review Board Approval (cdc.gov).

**Fig. 1:**
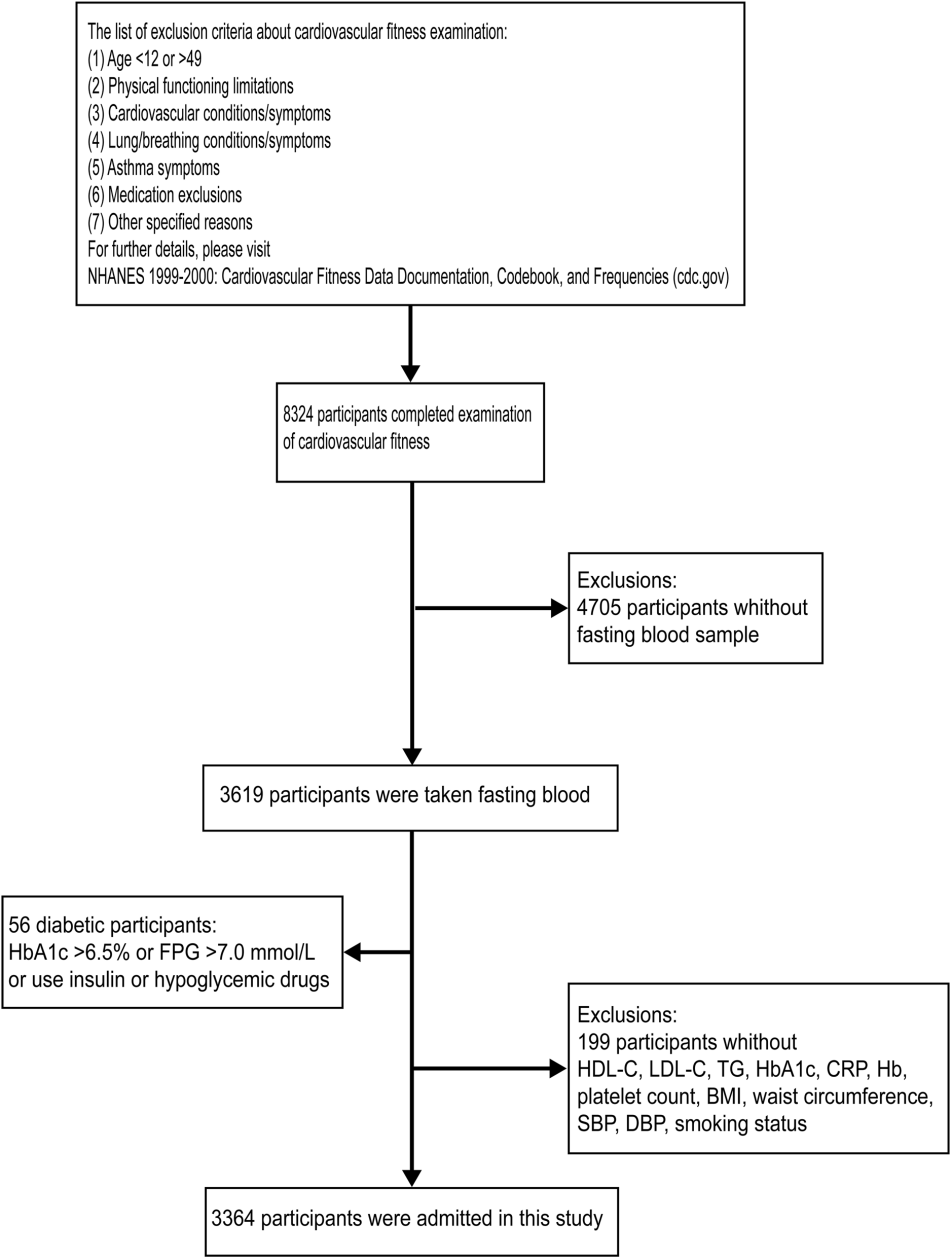
Flowchart of participant inclusion process. The list of exclusion criteria for CVF examination is available on NHANES 1999–2000: Cardiovascular Fitness Data Documentation, Codebook, and Frequencies (cdc.gov)

We used data from the NHANES study (1999–2004) to conduct a cross-sectional study of 3619 participants who completed CVF examination and had fasting blood samples taken. Only 12–49-year-old survey participants were eligible for the CVF examination. In CVF examination, participants were commonly directed to perform aerobic exercise on a treadmill. The list of exclusion criteria for CVF examination is available on NHANES 1999–2000: Cardiovascular Fitness Data Documentation, Codebook, and Frequencies (cdc.gov). The exclusion criteria of this study were as follows: (1) diabetic population: diabetic population were defined as glycosylated hemoglobin (HbA1c) ≥ 6.5% or fasting plasma glucose (FPG) ≥ 7.0 mmol/L [16]. Meanwhile, people who used insulin or hypoglycemic drugs were also defined as the diabetic population. (2) Participants with missing data. Finally, a total of 3364 participants were admitted to this study (Fig. 1). The study design for the survival cohort was shown in Additional file 1: Table S6.

### Definition of impaired CVF and exposure variable

NHANES categorized CVF into low, moderate and high levels. The levels of CVF were categorized according to sex- and age-specific cutoffs based on Vo2max. Cutoffs for adults aged 20–49 were determined from data in the Aerobics Center Longitudinal Study (ACLS), while standards for young individuals aged 12–19 aligned with those used in the FITNESSGRAM program (For further details, please refer to Additional file 3: Appendix I in NHANES Cardiovascular MEC Manual (cdc.gov)). The detailed procedure for estimating Vo2max can be found in Additional file 2: Appendix G of NHANES Cardiovascular MEC Manual (cdc.gov). Impaired CVF was defined as low and moderate CVF levels. NHANES used an autoanalyzer to enzymatically measure plasma triglyceride (TG) and FPG levels from fasting blood samples, which were taken from people fasting for at least 8 h or more but less than 24 h (The detailed measurement methods for laboratory indicators were outlined in Additional file 1: Table S3). The TyG index was calculated by $Ln[TG(mg/dL)\times FPG(mg/dl)/2]$[17].

#### Definition of covariates

Age, sex and race were acquired from demographic data. Body mass index (BMI), waist circumference, systolic blood pressure (SBP) and diastolic blood pressure (DBP) were acquired from examination data. To reduce errors, the average of the first three measurements was used instead of SBP and DBP.

C-reactive protein (CRP), hemoglobin (Hb), platelet count, low-density lipoprotein cholesterol (LDL-C), high-density lipoprotein cholesterol (HDL-C), HbA1c, FPG and TG were acquired from laboratory data.

Physical activity was transformed from “Physical activity readiness code” in the CVF examination, and we merged “Participating regularly in heavy physical activity for less than 30 min per week” and “Participating regularly in heavy physical activity for 30–60 min per week” into “Participating regularly in heavy physical activity less than 1 h per week”. Detailed definitions of levels 0 to 6 of physical activity can be found in Additional file 1: Table S1. We defined smoking status as "Current smoker", "Former smoker" and "Never smoker" (Detailed definitions of smoking status can be found in Additional file 1: Table S2).

The diagnosis of metabolic syndrome was established when meeting at least three of the following criteria [18]: (1) waist circumference > 102 cm in males or > 88 cm in females; (2) TG ≥ 150 mg/dL; (3) HDL-C < 40 mg/dL in males or < 50 mg/dL in females; (4) SBP ≥ 130 mmHg or DBP ≥ 85 mmHg; (5) FPG ≥ 110 mg/dL.

### Statistical analysis

The statistical analysis in this study were conducted by using R, version 4.3.1 (R Foundation). The “survey” package was utilized to account for the complex survey design in NHANES. Statistical significance was defined as a two-sided *p*-value of less than 0.05.

Population-weighted descriptive statistics were computed for the entire study population and categorized into quartiles based on the TyG index. Continuous variables were presented as medians with interquartile ranges (25th to 75th percentiles), while categorical variables were presented as unweighted frequencies along with weighted percentages. Differences between multiple groups of continuous variables were assessed using the Kruskal‒Wallis rank-sum test, and chi-squared tests were employed to assess significant differences between categorical variables.

Due to varying thresholds for determining impaired CVF across different age and sex groups, we included age and sex as covariates in each model. Subsequently, we introduced every variable into the weighted logistic regression model respectively. We assessed the association between each variable and impaired CVF after adjusting for age and sex. We then selected variables with* p* values less than 0.05 and included them as covariates in model 2. Model 3 adjusted for statistically significant and potentially clinically relevant variables. Model 1 adjusted for age, sex, and race. Model 2 adjusted for age, sex, and variables with *p* values < 0.05 after controlling for age and sex in the weighted logistic regressions. These variables included race, SBP, DBP, BMI, waist circumference, physical activity, metabolic syndrome, Hb, and HbA1c. Model 3 adjusted for statistically significant and potentially clinically relevant variables, including age, sex, race, BMI, waist circumference, physical activity, smoking status, metabolic syndrome, SBP, DBP, HbA1c, LDL-C, HDL-C, CRP, Hb, and platelet count. In addition, we performed a multicollinearity test for model 3 (Additional file 1: Table S5). The TyG index was included in the models, as a continuous variable or a categorical variable respectively. In addition, we explored the association between the TyG index and all-cause and cardiovascular mortality.

To further explore the relationship between the TyG index and impaired CVF, restricted cubic splines (RCS) with weight were employed (the calculation of weight in RCS was shown in Additional file 1: Table S4). Subgroup analysis was conducted based on sex, race, and metabolic syndrome status. Additionally, the population was stratified by age (less than 20 years) for further subgroup analysis. Mediation analysis was utilized to investigate whether certain variables act as mediators in the association between the TyG index and impaired CVF. Considering the simplicity and fitting performance of the model, we chose to retain only statistically significant continuous variables in model 2, excluding the TyG index, as mediating variables, including DBP, waist circumference and Hb. The other variables, age and sex were treated as covariates for conducting the mediation analysis. To test the robustness of the results, we employed the "jomo" package for imputing missing data and subsequently compared the results of RCS between the imputed dataset and the dataset with directly removed missing values. Additionally, we relaxed the exclusion criteria for participants with diabetes, excluding only those with FPG ≥ 7.0 mmol/L or HbA1c ≥ 6.5%. Subsequently, we conducted a comparative analysis of RCS results between the dataset with directly removed missing values and the imputed dataset.

## Results

### Study population

A total of 3364 participants were included in this study (Fig. 1). The study population with a median age of 28 (interquartile range: 19–37) years, among them, 1777 (51.80%) participants were male, and 1168 (67.51%) participants were non-Hispanic White participants. One-fifth of the population were current smokers, and one-tenth of the population had metabolic syndrome (Table 1).

**Table 1:** Baseline characteristics of the study population

Characteristic	Overall, N = 3364^a^
Age, years	28.00 (19.00–37.00)
Sex	
Female	1,587 (48.20)
Male	1,777 (51.80)
Race	
Mexican American	1,036 (10.10)
Non-Hispanic Black participants	886 (12.02)
Non-Hispanic White participants	1,168 (67.51)
Other Race	274 (10.38)
BMI, kg/m^2^	25.69 ± 5.70
Waist circumference, cm	88.07 ± 14.64
SBP, mmHg	113.24 ± 11.76
DBP, mmHg	68.56 ± 10.43
Physical activity	
level 0	302 (9.33)
level 1	529 (16.15)
level 2	669 (18.82)
level 3	1,107 (37.22)
level 4	118 (1.61)
level 5	208 (4.88)
level 6	431 (11.98)
Smoking status	
Never smoker	2,271 (61.92)
Former smoker	442 (14.99)
Current smoker	651 (23.08)
Metabolic syndrome	226 (9.07)
CRP, mg/dL	0.10 (0.04–0.27)
Hb, g/dL	14.59 ± 1.44
Platelet count, 10^9^ /L	268.26 ± 61.41
LDL, mmol/L	2.81 ± 0.86
HDL, mmol/L	1.32 ± 0.36
HbA1c, %	5.15 ± 0.29
FPG, mmol/L	5.12 ± 0.45
TG, mmol/L	1.02 (0.72–1.49)
Vo2max, ml/kg/min	40.89 ± 9.53
Levels of CVF	
Low level	924 (20.21)
Moderate level	1,285 (35.48)
High level	1,155 (44.30)
Impaired CVF	2,209 (55.70)
TyG index	8.36 ± 0.52
Standardization of TyG index	0.03 ± 0.16

^a^Median (25%-75%); n (unweighted) (%); Mean ± SD

*BMI* body mass index, *SBP* systolic blood pressure, *DBP* diastolic blood pressure, *CRP C*-reactive protein, *Hb* Hemoglobin, *LDL-C* low-density lipoprotein cholesterol, *HDL-C* high-density lipoprotein cholesterol, *HbA1c* glycohemoglobin, FPG fasting plasma glucose, *TG* triglycerides, Vo2max maximal oxygen consumption, *CVF* cardiovascular fitness, *TyG* index triglyceride-glucose index

The baseline information of the population included in the survival cohort was shown in Additional file 1: Table S7. Table 2 presented participant baseline characteristics by quantiles of the TyG index (Q1 (the lowest quartile): TyG index ≤ 7.97; Q2 (the second quartile): 7.97 < TyG index ≤ 8.33; Q3 (the upper quartile): 8.33 < TyG index ≤ 8.72; Q4 (the highest quartile): TyG index > 8.72). Compared with participants in the low TyG index group, participants in the high TyG index group were usually elderly, male, Mexican American or non-Hispanic White participants, had high BMI, waist circumference, SBP, DBP, CRP, Hb, platelet count, LDL-C, HbA1c, FPG and TG, and low levels of physical activity, HDL-C and Vo2max. Due to the close association between impaired CVF and age and sex, we wound further analyze the relationship between impaired CVF and the TyG index after adjusting for age and sex.

**Table 2:** Baseline characteristics of the study population according to the quartiles of the TyG index

Characteristic	Q1, N = 1086^a^	Q2, N = 904^a^	Q3, N = 737^a^	Q4, N = 637 ^1^	*p*-value^b^
Age, years	22.00 (17.00–33.00)	26.00 (17.00–36.00)	30.00 (20.00–38.00)	32.00 (23.00–40.93)	** < 0.001**
Sex					** < 0.001**
Female	557 (53.98)	480 (58.49)	322 (44.60)	228 (35.73)	
Male	529 (46.02)	424 (41.51)	415 (55.40)	409 (64.27)	
Race					** < 0.001**
Mexican American	260 (7.21)	284 (9.62)	261 (10.85)	231 (12.70)	
Non-Hispanic Black participants	442 (20.58)	241 (13.01)	127 (8.46)	76 (6.03)	
Non-Hispanic White participants	302 (62.76)	301 (66.29)	291 (70.49)	274 (70.49)	
Other Race	82 (NA)	78 (NA)	58 (NA)	56 (NA)	
BMI, kg/m^a^	22.78 (20.25–25.35)	23.58 (20.82–27.21)	25.05 (22.14–29.56)	27.93 (24.71–31.09)	** < 0.001**
Waist circumference, cm	79.90 (72.80–88.30)	81.13 (74.40–91.61)	88.38 (79.80–99.70)	96.60 (87.88–105.24)	** < 0.001**
SBP, mmHg	109.33 (103.33–117.33)	110.00 (103.33–118.00)	114.00 (107.33–121.33)	116.00 (108.67–123.33)	** < 0.001**
DBP, mmHg	66.00 (59.33–72.00)	68.00 (61.33–73.33)	70.00 (63.33–76.00)	72.00 (65.33–78.00)	** < 0.001**
Physical activity					**0.041**
level 0	83 (7.69)	76 (7.02)	69 (10.70)	74 (11.92)	
level 1	165 (14.29)	148 (18.75)	112 (16.44)	104 (15.10)	
level 2	197 (17.39)	181 (16.70)	151 (20.28)	140 (20.94)	
level 3	348 (36.88)	284 (37.07)	247 (35.87)	228 (39.07)	
level 4	41 (1.54)	35 (2.34)	30 (1.43)	12 (1.14)	
level 5	78 (7.13)	63 (5.14)	48 (5.22)	19 (2.04)	
level 6	174 (15.08)	117 (12.99)	80 (10.06)	60 (9.80)	
Smoking status					**0.007**
Never smoker	797 (67.57)	640 (66.52)	462 (57.56)	372 (56.04)	
Former smoker	116 (11.92)	107 (13.27)	111 (16.87)	108 (17.93)	
Current smoker	173 (20.51)	157 (20.22)	164 (25.57)	157 (26.03)	
Metabolic syndrome	4 (0.32)	12 (1.74)	20 (4.35)	190 (29.89)	** < 0.001**
CRP, mg/dL	0.05 (0.03–0.16)	0.07 (0.03–0.24)	0.12 (0.05–0.32)	0.19 (0.08–0.42)	** < 0.001**
Hb, g/dL	14.30 (13.30–15.20)	14.20 (13.40–15.20)	14.90 (13.80–15.80)	15.20 (14.10–16.00)	** < 0.001**
Platelet count, 10^9^ /L	253.00 (216.00–295.00)	258.00 (223.00–300.00)	269.24 (227.94–304.00)	268.97 (233.00–315.00)	**0.007**
LDL, mmol/L	2.30 (1.89–2.87)	2.61 (2.15–3.13)	2.95 (2.43–3.44)	3.15 (2.53–3.75)	** < 0.001**
HDL, mmol/L	1.42 (1.22–1.72)	1.34 (1.16–1.63)	1.22 (1.06–1.45)	1.09 (0.93–1.28)	** < 0.001**
HbA1c, %	5.10 (4.90–5.30)	5.10 (5.00–5.30)	5.20 (5.00–5.35)	5.20 (5.00–5.40)	** < 0.001**
FPG, mmol/L	4.92 (4.67–5.21)	5.03 (4.79–5.32)	5.15 (4.92–5.48)	5.30 (4.97–5.61)	** < 0.001**
TG, mmol/L	0.60 (0.51–0.67)	0.88 (0.79–0.95)	1.21 (1.10–1.35)	1.94 (1.68–2.34)	** < 0.001**
Vo2max, ml/kg/min	40.81 (34.56–47.28)	39.93 (34.17–46.84)	38.93 (34.25–45.23)	39.52 (34.38–44.73)	**0.028**
Impaired CVF	718 (52.99)	595 (55.05)	493 (58.93)	403 (55.82)	0.321
TyG index	7.76 (7.61–7.88)	8.16 (8.07–8.25)	8.51 (8.43–8.61)	9.00 (8.84–9.20)	** < 0.001**
Standardization of TyG index	-0.15 (-0.20–0.12)	-0.03 (-0.06–0.00)	0.08 (0.05–0.11)	0.23 (0.18–0.30)	** < 0.001**

^a^Median (25%-75%); n (unweighted) (%)

^b^Kruskal-Wallis rank-sum test for complex survey samples; chi-squared test with Rao & Scott's second-order correction

*BMI* body mass index, *SBP* systolic blood pressure, *DBP* diastolic blood pressure, *CRP C*-reactive protein, *Hb* Hemoglobin, *LDL-C* low-density lipoprotein cholesterol, *HDL-C* high-density lipoprotein cholesterol, *HbA1c* glycohemoglobin, *FPG* fasting plasma glucose, *TG* triglycerides, *Vo2max* maximal oxygen consumption, *CVF* cardiovascular fitness, *TyG* index triglyceride-glucose index

*p* values in bold are < 0.05

### TyG index and impaired CVF

To eliminate the effects of age and sex on impaired CVF, age and sex were included as covariables in the weighted logistic model. As presented in Table 3, non-Hispanic Black participants, BMI, waist circumference, SBP, DBP, metabolic syndrome, HbA1c, FPG, TG and TyG index had a positive correlation with impaired CVF. Hb and level 6 physical activity had a negative correlation with impaired CVF. In three weighted multivariable logistic models, whether increased by 1 unit or 1 SD, the TyG index was significantly and positively correlated with impaired CVF. The TyG index was further categorized into quartiles, and the Q1 group was used as a reference to evaluate the association between other quantile groups and impaired CVF. In model 3, compared with Q1, a significant increase in the odds ratio (OR) was found in Q3 (OR: 1.61; 95% Cl 1.21–2.13) and Q4 (OR: 1.55; 95% Cl 1.06–2.26), and the *p* for trend was 0.009 (Table 4). RCS analysis (Additional file 1: Figure S1) showed a dose‒response relationship between the TyG index and impaired CVF (*p* nonlinear = 0.290). Association between the TyG index and all-cause and cardiovascular mortality was shown in Additional file 1: Table S8. RCS analysis between the TyG index and all-cause and cardiovascular mortality was shown in Additional file 1: Figure S2 and S3.

**Table 3:** Association between different variables and impaired cardiovascular fitness after adjusting for age and sex

Variables	HR (95%CI)	*p*-value
Race		** < 0.001**
Mexican American	Reference	
Non-Hispanic Black participants	1.45 (1.10–1.92)	
Non-Hispanic White participants	0.84 (0.64–1.10)	
Other Race	1.18 (0.76–1.85)	
BMI	1.04 (1.02–1.06)	** < 0.001**
Waist circumference	1.02 (1.01–1.03)	** < 0.001**
SBP	1.01 (1.00–1.02)	**0.023**
DBP	1.02 (1.01–1.03)	** < 0.001**
Physical activity		** < 0.001**
level 0	Reference	
level 1	1.17 (0.76–1.79)	
level 2	1.30 (0.82–2.06)	
level 3	1.20 (0.79–1.82)	
level 4	0.86 (0.39–1.90)	
level 5	0.64 (0.36–1.63)	
level 6	0.46 (0.28–0.77)	
Smoking status		0.136
Never smoker	Reference	
Former smoker	1.11 (0.81–1.53)	
Current smoker	0.80 (0.62–1.03)	
Metabolic syndrome	1.50 (1.06–2.14)	**0.019**
CRP	1.01 (0.80–1.27)	0.925
Hb	0.89 (0.82–0.96)	**0.002**
Platelet count	1.00 (1.00–1.00)	0.096
LDL-C	1.13 (0.97–1.31)	0.095
HDL-C	0.82 (0.64–1.06)	0.118
HbA1c	1.85 (1.26–2.72)	**0.001**
TyG index	1.61 (1.32–1.95)	** < 0.001**

Due to varying thresholds for determining impaired CVF across different age groups and sex, we examined the relationship between each variable and impaired cardiovascular endurance after adjusting for age and sex

*BMI* body mass index, *SBP* systolic blood pressure, DBP diastolic blood pressure, CRP C-reactive protein, Hb Hemoglobin, LDL-C low-density lipoprotein cholesterol, HDL-C high-density lipoprotein cholesterol, HbA1c glycohemoglobin, TyG index triglyceride-glucose index

*p* values in bold are < 0.05

**Table 4:** Association between the TyG index and impaired cardiovascular fitness in different Logistic models

TyG index	HR (95%CI)		
	Model 1	Model 2	Model 3
Per 1 unit increase	1.60 (1.31–1.94)^**^	1.39 (1.11–1.74)^*^	1.46 (1.13–1.90) ^*^
Per 1 SD increase	4.46 (2.38–8.35)^**^	2.84 (1.38–5.83)^*^	3.38 (1.47–7.76) ^*^
Q1	Reference	Reference	Reference
Q2	1.26(0.99–1.60)	1.22(0.94–1.57)	1.24 (0.96–1.60)
Q3	1.80 (1.35–2.38)^**^	1.53 (1.17–2.00)^*^	1.61 (1.21–2.13)^*^
Q4	1.81 (1.34–2.44)^**^	1.45 (1.02–2.06)^*^	1.55 (1.06–2.26)^*^
*p* for trend	** < 0.001**	**0.011**	**0.009**

Model 1 was adjusted for age, sex, and race

Model 2 adjusted for age, sex, and variables with P < 0.05 after controlling for age and sex in the weighted logistic regressions. These variables included race, SBP, DBP, *BMI*, waist circumference, physical activity, metabolic syndrome, *Hb*, and HbA1c

Model 3 adjusted for all variables, including age, sex, race, BMI, waist circumference, physical activity, smoking status, metabolic syndrome, SBP, DBP, HbA1c, LDL-C, HDL-C, CRP, Hb, and platelet count

*p* values in bold are < 0.05

^*^ *p* < 0.05

^**^ *p* < 0.001

### Subgroup analysis

**Fig. 2:**
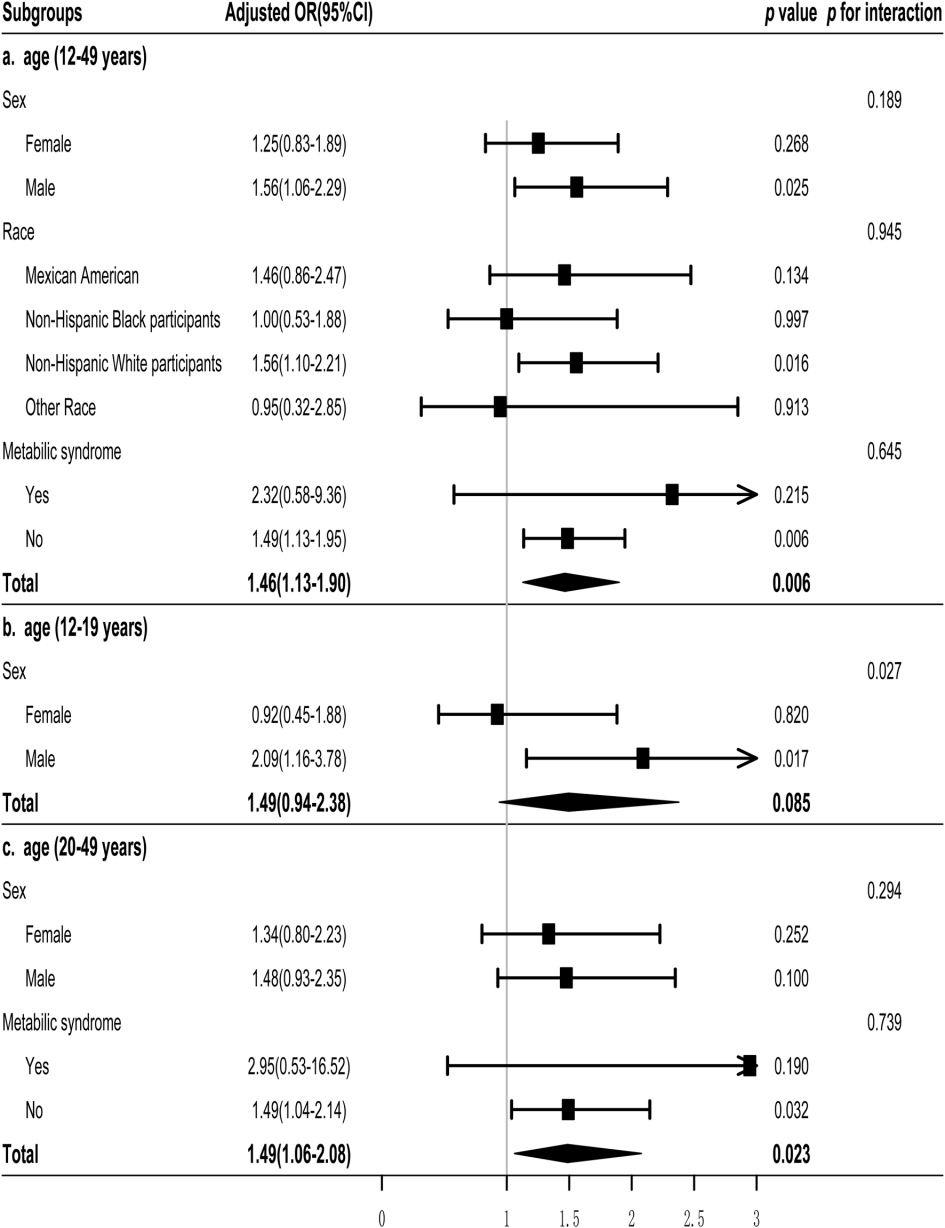
Subgroup and interaction analyses of the TyG index and impaired cardiovascular fitness. Multivariable weighted logistic model adjusted for all variables, including age, sex, race, BMI, waist circumference, physical activity, smoking status, metabolic syndrome, SBP, DBP, HbA1c, LDL-C, HDL-C, CRP, Hb, and platelet count

To further investigate the association between the TyG index and impaired CVF across diverse population, we stratified the population based on sex, race, and the presence of metabolic syndrome. Weighted multivariable logistic regression analysis presented no significant interactions between sex, race, metabolic syndrome, and the TyG index for impaired CVF (all *p* for interaction ≥ 0.189). Among males, non-Hispanic White participants, and individuals without metabolic syndrome, the TyG index displayed a significant association with impaired CVF (Fig. 2a).

We further subdivided the population based on age, distinguishing those older than 20 years from those younger. In the population under 20 years old, due to a limited number of cases with metabolic syndrome and other race, metabolic syndrome and race were not considered as subgroup criteria. Similarly, among individuals aged 20 years and older, race was not considered as a subgroup criterion.

Among individuals aged less than 20 years, although the association between the TyG index and impaired CVF was not significant in the overall population, a significant association was observed among males. Furthermore, a significant interaction (*p* = 0.027) between sex and the TyG index for impaired CVF was noted (Fig. 2b).

Among participants aged 20 years and older, the TyG index exhibited a significant association with impaired CVF (adjusted OR and 95% CI: 1.49 and 1.06–2.08). However, this significance was only observed in participants without metabolic syndrome (Fig. 2c).

### Mediation analysis

**Fig. 3:**
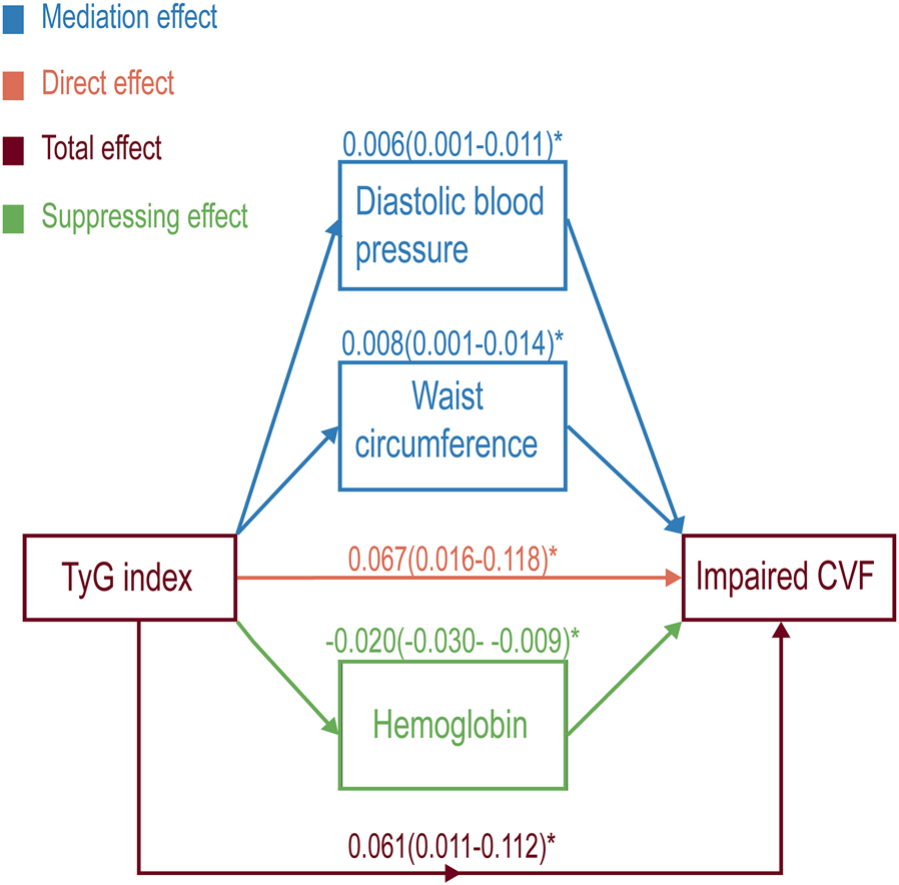
Mediation analysis. Age, sex, race, physical activity, metabolic syndrome, SBP and BMI were included as covariate variables. *p < 0.05

Age, sex, race, physical activity, metabolic syndrome, SBP and BMI were included as covariates, while DBP, waist circumference and Hb were identified as mediating variables to explore their influence on the association between the TyG index and impaired CVF. The mediation effect of DBP was 0.006(0.001–0.011) and the mediation effect of waist circumference was 0.008(0.001–0.014). Hb exerted a suppressing effect on the relationship between the TyG indx and impaired CVF, with a value of -0.020(-0.030–-0.009). The *p* values for all the above effects were less than 0.05 (Fig. 3).

### Sensitivity analysis

The results of the sensitivity analysis were as follows: (1) When evaluated through restricted cubic splines, the results from imputed dataset were found to be consistent with the results obtained from the dataset with directly removed missing values (see Additional file 1: Figure S4). (2) Excluding individuals with FPG ≥ 7.0 mmol/L or HbA1c ≥ 6.5%, the RCS analysis results showed no significant difference between imputed dataset and the dataset with directly removed missing values (see Additional file 1: Figure S5).

## Discussion

Based on the 1999–2004 NHANES database, we first revealed the association between the TyG index and impaired CVF in the young non-diabetic population, filling the gap in research on the TyG index in young non-diabetic people. Moreover, we found a significant interaction of sex on the relationship between the TyG index and impaired CVF in the population with age less than 20 years.

Previous studies have shown that the TyG index is associated with coronary artery calcification, subclinical atherosclerosis, and arterial stiffness [19–21]. These results indicated a close relationship between the TyG index and the incidence of CVD. However, the research population about the TyG index and CVD were mainly middle-aged and elderly individuals [11, 12]. Considering the lower prevalence of CVD and diabetes in the young population, we chose to utilize impaired CVF instead of CVD among non-diabetic young individuals to examine the relationship between the TyG index and impaired CVF. Our current study found that individuals with elevated TyG index were more likely to experience impaired CVF in this non-diabetic young population. Nevertheless, the effect of the TyG index on CVF in diabetic population is still to be elucidated.

In this study, we did not find statistically significant association between the TyG index and all-cause and cardiovascular mortality, which might be due to the fact that our study population was young and had no comorbid cardiovascular or other underlying diseases, leading to low mortality during the follow up. Future long-term prospective studies are needed to further investigate the impact of the baseline TyG index on impaired CVF and subsequent cardiovascular events in young non-diabetic population.

A meta-analysis of 87 studies indicated that females with metabolic syndrome have a higher risk of CVD than males [22]. Moreover, among population with impaired glucose tolerance, females have a higher risk of coronary heart disease than males [23]. In this way, compared with males, females are at high risk of CVD. However, in our research, we only found a significant association between the TyG index and impaired CVF in males. The reason for this difference may be that most females in previous studies were in late perimenopause or menopause. Endogenous estrogen can improve insulin sensitivity in females in a rodent model [24]. In addition, estradiol reduced insulin resistance, modifying both endothelium-dependent and calcium-dependent processes [25]. By comparing the cardiovascular risk factors for premenopausal and postmenopausal women of the same age, H W Peters and colleagues found that TC, LDL-C and apolipoprotein B increased significantly in postmenopausal women [26]. These results to some extent explained the phenomenon that females have higher cardiovascular risk factors than males in previous studies. The age of our study population was 28 (interquartile range: 19–37) years old. Compared with middle-aged and elderly females, females in this age group have plenty of estrogen to reduce insulin resistance, which may explain why our results are different from previous studies. Growth hormone levels are higher in women under 20 years of age than in adult women [27]. Previous studies have shown that growth hormone can provide cardiovascular protection and improve cardiovascular function [28–30]. This may be the reason for the significant interaction between sex and the TyG index for impaired CVF only in people below 20 years old.

Metabolic syndrome is a cardiovascular risk factor [22, 31]. No statistical significance between the TyG index and impaired CVF was found in the population with metabolic syndrome. First, the use of lipid-lowering drugs can affect the levels of TG, causing the TyG index to be lower than normal. On the other hand, strengthening exercise, as an important measure to interfere with metabolic syndrome, can increase CRF [32, 33].

It was noteworthy that our study was conducted with a group of non-diabetic young participants twenty years ago. According to the 2020 epidemiological survey on metabolic syndrome in the United States, a significant increase of metabolic syndrome was observed among young adults [34]. Thus, although our present results did not find significant association between the TyG index and impaired CVF in participants with metabolic syndrome, this association should be reconfirmed in contemporary data.

The results of intermediary analysis suggested that part of the harmful effects of the TyG index on CVF were realized through its effects on blood pressure and abdominal obesity. Simultaneously, the TyG index enhanced the oxygen transport capacity by increasing Hb, thereby enhancing Vo2max, which was utilized for assessing CVF. There was a lack of dedicated research exploring the correlation between the TyG index and Hb. However, earlier studies suggested that individuals with structural anomalies in Hb typically exhibited lower insulin resistance compared to those with normal Hb levels [35]. This finding might have constituted a supporting element for the outcomes of our intermediary analysis. However, further research is necessary to substantiate the potential relationship between the TyG index and Hb.

Previous studies have revealed the role of the TyG index in the occurrence, development and prognosis of CVD [36–38]. In our study, we found that an increase in the TyG index can reduce the level of CVF in non-diabetic young population, especially among males. Because the reduction in CVF is associated with an increase in cardiovascular mortality and all-cause mortality [4, 5], we suggested that young people should pay attention to controlling the TyG index to improve CVF and reduce the incidence of CVD.

## Limitations

The study had several notable limitations. First, due to the cross-sectional design of our study, the conclusions drawn need to be validated by prospective studies. Although we performed a correlation analysis involving the TyG index with all-cause and cardiovascular mortality, we did not perform a correlation analysis of the TyG index with adverse cardiovascular events because of limitations in the NHANES database. Second, previous studies suggested that alcohol consumption and smoking impacted Vo2max [39, 40]. However, because the NHANES database lacked questionnaire data on underage drinking between 1999 and 2004, we excluded alcohol use as a variable in our study. Similarly, specific information on the number of cigarettes smoked by participants and the duration of their smoking history was not available due to database limitations, thus limiting the interpretation of smoking status. In addition, the diagnostic criteria for diabetes mellitus in this study did not include oral glucose tolerance test, which may underestimate the prevalence of these conditions among the participants [41, 42]. Moreover, the use of submaximal treadmill testing to measure Vo2max in NHANES, which is less accurate than maximal treadmill testing, might affect the study results [43, 44]. Finally, the generalizability of this study to other countries or population was limited by the fact that the participants were predominantly from the United States.

## Conclusions

In non-diabetic young population, individuals with higher TyG index values are more likely to experience impaired CVF. Additionally, sex may exert an influence on CVF, with males being more susceptible to impaired CVF under similar TyG index conditions.

### Supplementary Information

**Additional file1: Table S1.** Definition of physical activity. **Table S2.** Definition of smoking status. **Table S3.** The measurement methods of laboratory indicators. **Table S4.**The weights used in the analysis of this study. **Table S5.** The collinearity assessment outcomes for Model 3. **Table S6.** Comprehensive details of the Survival Cohort Study Design for NHANES 1999-2004. **Table S7.** Baseline characteristics of the study population with accessible survival outcomes. **Table S8.** Hazard ratios for all-cause mortality and cardiovascular mortality according to TyG Index. **Figure S1.** The association between TyG index and CVF impairment. **Figure S2.** The association between TyG index and all-cause mortality. Figure S3 The association between TyG index and cardiovascular mortality. **Figure S4.** Sensitivity analysis 1. **Figure S5.** Sensitivity analysis 2.Done

**Additional file 2: Appendix G.** Calculation Of Estimated Vo2 Max.

**Additional file 3. Appendix I.** Reference ranges for vo2 max.

## Data Availability

The datasets analyzed during the current study are available in the NHANES - National Health and Nutrition Examination Survey Homepage (cdc.gov).

## Abbreviations

TyG index: Triglyceride-glucose index
CVD: Cardiovascular disease
NHANES: National Health and Nutrition Examination Survey
CVF: Cardiovascular fitness
CRF: Cardiorespiratory fitness
RCS: Restricted cubic splines
OR: Odds ratio
CI: Confidence interval
HbA1c: Glycosylated hemoglobin
FPG: Fasting plasma glucose
TG: Triglyceride
BMI: Body mass index
SBP: Systolic blood pressure
DBP: Diastolic blood pressure
CRP: C-reactive protein
Hb: Hemoglobin
LDL-C: Low-density lipoprotein cholesterol
HDL-C: High-density lipoprotein cholesterol
