# Supplementary material for: Association between the triglyceride-glucose index and impaired cardiovascular fitness in non-diabetic young population
Source: Cardiovasc Diabetol. 2024 Jan 20;23:39. doi: 10.1186/s12933-023-02089-8 (PMC10800072; doi:10.1186/s12933-023-02089-8)
Supplement: Supplementary file 1 — Additional file1: Table S1. Definition of physical activity. Table S2. Definition of smoking status. Table S3. The measurement methods of laboratory indicators. Table S4.The weights used in the analysis of this study. Table S5. The collinearity assessment outcomes for Model 3. Table S6. Comprehensive details of the Survival Cohort Study Design for NHANES 1999-2004. Table S7. Baseline characteristics of the study population with accessible survival outcomes. Table S8. Hazard ratios for all-cause mortality and cardiovascular mortality according to TyG Index. Figure S1. The association between TyG index and CVF impairment. Figure S2. The association between TyG index and all-cause mortality. Figure S3 The association between TyG index and cardiovascular mortality. Figure S4. Sensitivity analysis 1. Figure S5. Sensitivity analysis 2.Done [file 12933_2023_2089_MOESM1_ESM.docx]

**Table S1 Definition of physical activity**

**Table S2 Definition of smoking status**

**Table S3** **The measurement methods of laboratory indicators**

**Table S4 The weights used in the analysis of this study**

**Table S5 The collinearity assessment outcomes for Model 3**

**Table S6 Comprehensive details of the Survival Cohort Study Design for NHANES 1999-2004**

**Table S7 Baseline characteristics of the study population with accessible survival outcomes**

**Table S8 Hazard ratios for all-cause mortality and cardiovascular mortality according to TyG Index**

**Figure S1 The association between TyG index and CVF impairment**

**Figure S2 The association between TyG index and all-cause mortality**

**Figure S3 The association between TyG index and cardiovascular mortality**

**Figure S4 Sensitivity analysis 1**

**Figure S5 Sensitivity analysis 2**

| **Table S1 Definition of physical activity** | |
| --- | --- |
| Level 0 | Little or no regular recreation, sport or physical activity and avoids walking or exertion |
| Level 1 | Little or no regular recreation, sport or physical activity but walks for pleasure and occasionally exercises |
| Level 2 | participating regularly in recreation or work requiring modest physical activity for 10 to 60 minutes per week |
| Level 3 | participating regularly in recreation or work requiring modest physical activity for more than 60 minutes per week |
| Level 4 | participating regularly in heavy physical activity less than 1 hour per week |
| Level 5 | participating regularly in heavy physical activity for 1 - 3 hours per week |
| Level 6 | participating regularly in heavy physical activity for more than 3 hours per week |
| A series of questions to describe the participant's typical physical activity level. (See [NHANES Cardiovascular MEC Manual (cdc.gov)](https://wwwn.cdc.gov/nchs/data/nhanes/2003-2004/manuals/cv_99-04.pdf), Appendix E) | |

| **Table S2 Definition of smoking status** | |
| --- | --- |
| 12-19 years |  |
| Current smokers | Participants had smoked a full cigarette and continued smoking in the past month |
| Former smokers | Participants had smoked a full cigarette but did not smoke in the past month |
| Never smokers | Participants had not smoked a full cigarette or had never tried smoking |
| 20-49 years |  |
| Current smokers | Participants had smoked more than 100 cigarettes in their lifetime and currently smoking |
| Former smokers | Participants had smoked more than 100 cigarettes in their lifetime but not smoking recently |
| Never smokers | Participants had smoked fewer than 100 cigarettes in their lifetime |

| **Table S3** **The measurement methods of laboratory indicators** | |
| --- | --- |
| NHANES Laboratory Procedures Manual | [1999-2000 MEC Laboratory/Medical Technologists Procedures Manual (cdc.gov)](https://wwwn.cdc.gov/nchs/data/nhanes/1999-2000/manuals/1999-2000_MEC_Laboratory_Manual.pdf) |
| LDL-C | [NHANES 1999-2000: Cholesterol - LDL & Triglycerides Data Documentation, Codebook, and Frequencies (cdc.gov)](https://wwwn.cdc.gov/Nchs/Nhanes/1999-2000/LAB13AM.htm) |
| TG |  |
| FPG | [NHANES 1999-2000: Plasma Fasting Glucose, Serum C-peptide & Insulin Data Documentation, Codebook, and Frequencies (cdc.gov)](https://wwwn.cdc.gov/Nchs/Nhanes/1999-2000/LAB10AM.htm) |
| CRP | [NHANES 1999-2000: C-Reactive Protein (CRP) Data Documentation, Codebook, and Frequencies (cdc.gov)](https://wwwn.cdc.gov/Nchs/Nhanes/1999-2000/LAB11.htm) |
| Platelet count | [NHANES 1999-2000: Complete Blood Count with 5-part Differential - Whole Blood Data Documentation, Codebook, and Frequencies (cdc.gov)](https://wwwn.cdc.gov/Nchs/Nhanes/1999-2000/LAB25.htm) |
| Hb |  |
| HbA1c | [NHANES 1999-2000: Glycohemoglobin Data Documentation, Codebook, and Frequencies (cdc.gov)](https://wwwn.cdc.gov/Nchs/Nhanes/1999-2000/LAB10.htm) |
| HDL-C | [NHANES 1999-2000: Cholesterol - Total & HDL Data Documentation, Codebook, and Frequencies (cdc.gov)](https://wwwn.cdc.gov/Nchs/Nhanes/1999-2000/LAB13.htm) |

For ease of reference, detailed descriptions from the NHANES official website regarding laboratory indicator measurement methods have been compiled in the table below, using data from the 1999-2000 period as an example (
The laboratory examination methods remained consistent across the three periods: 1999-2000, 2001-2002, and 2003-2004)

| **Table S4 The weights used in the analysis of this study** | |
| --- | --- |
| **Weight** | **Calculation method** |
| Weights used in descriptive statistics  and logistic regression | Weights=2*WTSAF4YR/3+WTSAF2YR/3 |
| Weights used in RCS | R code:  ori.weight<1/(Model3$survey.design$prob)  mean.weight<-mean(ori.weight)  data$weight<ori.weight/mean.weight |

WTSAF4YR: 4-year fasting sample weight from 1999 to 2002

WTSAF2YR: 2-year fasting sample weight from 2003 to 2004

Model3: Weighted Logistic regression after adjustment of all variables

| **Table S5 The collinearity assessment outcomes for Model 3** | | | |
| --- | --- | --- | --- |
| variables | GVIF | df | GVIF^(1/(2*df)) |
| TyG index | 3.10 | 1 | 1.76 |
| Age, years | 3.03 | 1 | 1.74 |
| Sex | 6.75 | 1 | 2.60 |
| Race | 22.79 | 3 | 1.68 |
| LDL-C, mmol/L | 4.32 | 1 | 2.08 |
| HDL-C, mmol/L | 3.74 | 1 | 1.93 |
| CRP, mg/dL | 2.76 | 1 | 1.66 |
| HbA1c, % | 2.24 | 1 | 1.50 |
| Hb, g/dL | 5.39 | 1 | 2.32 |
| Platelet count, 10^9^ /L | 1.60 | 1 | 1.26 |
| Physical activity | 153.25 | 6 | 1.52 |
| SBP, mmHg | 3.13 | 1 | 1.77 |
| DBP, mmHg | 2.14 | 1 | 1.46 |
| Metabolic syndrome | 3.23 | 1 | 1.80 |
| Smoking status | 11.28 | 2 | 1.83 |
| BMI, kg/m2 | 21.33 | 1 | 4.62 |
| Waist circumference, cm | 19.69 | 1 | 4.44 |

BMI body mass index, SBP systolic blood pressure, DBP diastolic blood pressure, CRP C-reactive protein, Hb Hemoglobin, LDL-C low-density lipoprotein cholesterol, HDL-C high-density lipoprotein cholesterol, HbA1c glycohemoglobin, TyG index triglyceride-glucose index

| **Table S6 Comprehensive Details of the Survival Cohort Study Design for NHANES 1999-2004** | |
| --- | --- |
| Study population | The non-diabetic population participating in the cardiovascular fitness examination. Due to the unavailability of survival data for individuals under the age of 18 in NHANES, the age range for the survival cohort study is restricted to 18-49 years. |
| Exclusion Criteria | [NHANES 1999-2000: Cardiovascular Fitness Data Documentation, Codebook, and Frequencies (cdc.gov)](https://wwwn.cdc.gov/Nchs/Nhanes/1999-2000/CVX.htm#Appendix_A._Classification_of_the_Exclusion_Criteria_in_the_NAHNES_Cardiovascular_Fitness_Component) |
| Definition of Mortality Outcome | We categorized all causes of death as all-cause mortality. |
|  | Cardiovascular mortality was defined by the International Classification of Diseases, 10th Revision (ICD-10) codes 054-064. |
| Source of Survival Data | Mortality outcomes were determined by linking the dataset to National Death Index (NDI) records up to December 31, 2019. [NCHS Data Linkage - Mortality Data - Public-Use Files (cdc.gov)](https://www.cdc.gov/nchs/data-linkage/mortality-public.htm) |
| Follow-up Status | In total, 1877 participants were enrolled for follow-up, up until December 31, 2019, with one participant lost to follow-up |

| **Table S7 Baseline Characteristics of the Study Population with Accessible Survival Outcomes** | |
| --- | --- |
| **Characteristic** | **Overall**, N = 1844 ^1^ |
| Age, years | 32.00 (24.00-39.00) |
| Sex |  |
| Female | 873 (47.99) |
| Male | 971 (52.01) |
| Race |  |
| Mexican American | 507 (9.87) |
| Non-Hispanic Black participants | 421 (11.49) |
| Non-Hispanic White participants | 773 (69.09) |
| Other Race | 143 (9.55) |
| BMI, kg/m^2^ | 26.53±5.56 |
| Waist circumference, cm | 90.57±13.98 |
| SBP, mmHg | 114.42±11.84 |
| DBP, mmHg | 70.40±9.63 |
| Physical activity |  |
| level 0 | 209 (10.50) |
| level 1 | 338 (17.49) |
| level 2 | 345 (18.51) |
| level 3 | 657 (38.54) |
| level 4 | 30 (0.82) |
| level 5 | 80 (4.13) |
| level 6 | 185 (10.00) |
| Smoking status |  |
| Never smoker | 1,053 (57.52) |
| Former smoker | 292 (16.31) |
| Current smoker | 499 (26.17) |
| Metabolic syndrome | 196 (10.89) |
| CRP, mg/dL | 0.13 (0.05-0.32) |
| Hb, g/dL | 14.66±1.47 |
| Platelet count, 10^9^ /L | 264.69±60.87 |
| LDL, mmol/L | 2.94±0.87 |
| HDL, mmol/L | 1.34±0.38 |
| HbA1c, % | 5.15±0.30 |
| FPG, mmol/L | 5.14±0.47 |
| TG, mmol/L | 1.07 (0.76-1.57) |
| TyG index | 8.42±0.53 |
| Standardization of TyG index | 0.01±0.18 |
| All-cause mortality | 52 (2.63) |
| Cardiovascular mortality | 11 (0.57) |
| ^1^Median (25%-75%); n (unweighted) (%); Mean ± SD | |

BMI body mass index, SBP systolic blood pressure, DBP diastolic blood pressure, CRP C-reactive protein, Hb Hemoglobin, LDL-C low-density lipoprotein cholesterol, HDL-C high-density lipoprotein cholesterol, HbA1c glycohemoglobin, FPG fasting plasma glucose, TG triglycerides, Vo2max maximal oxygen consumption, TyG index triglyceride-glucose index

| **Table S8 Hazard Ratios for All-cause Mortality and Cardiovascular Mortality According to TyG Index** | | |
| --- | --- | --- |
| TyG index | HR (95%CI), *p* value | |
|  | All-cause mortality | Cardiovascular mortality |
| Per 1 unit increase | 0.94(0.48-1.84), 0.853 | 2.79(0.66-11.7), 0.161 |
| Per 1 SD increase | 0.83(0.12-5.97), 0.853 | 20.5(0.30-1,402), 0.161 |
| Q1 | Reference | Reference |
| Q2 | 0.79(0.32-1.98), 0.618 | 3.52(0.15-83.0), 0.435 |
| Q3 | 2.51(0.94-6.68), 0.067 | 11.6(1.19-113), 0.035 |
| Q4 | 1.13(0.35-3.64), 0.834 | 14.4(0.77-271), 0.074 |
| p for trend | 0.413 | 0.075 |

In the exploration of the association between the TyG index and cardiovascular mortality, we observed significant collinearity between BMI and waist circumference. Consequently, we decided to exclude BMI as a covariate from the model.

The model adjusted for age, sex, race, waist circumference, physical activity, current smoking, metabolic syndrome, SBP, DBP, HbA1c, LDL-C, HDL-C, CRP, Hb, and platelet count.

**Figure S1 The association between TyG index and suboptimal CVH**


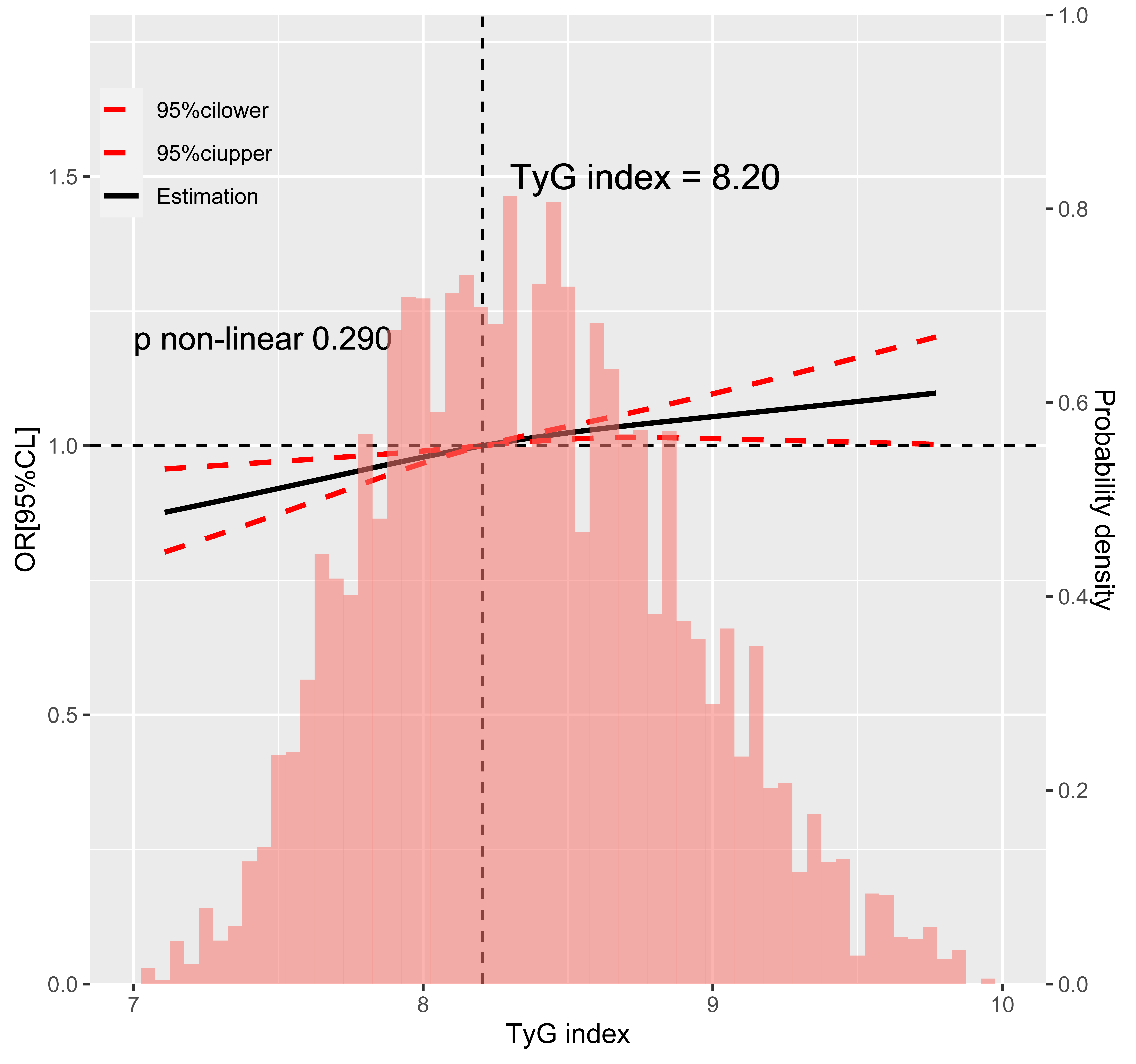


The data were analyzed by fitting logistic regression models with weights, utilizing a restricted cubic spline featuring three knots positioned at the 10th, 50th, and 90th percentiles of the baseline TyG index. The model adjusted for age, sex, race, BMI, waist circumference, physical activity, current smoking, metabolic syndrome, SBP, DBP, HbA1c, LDL-C, HDL-C, CRP, Hb, and platelet count.

**Figure S2 The association between TyG index and all-cause mortality**

**
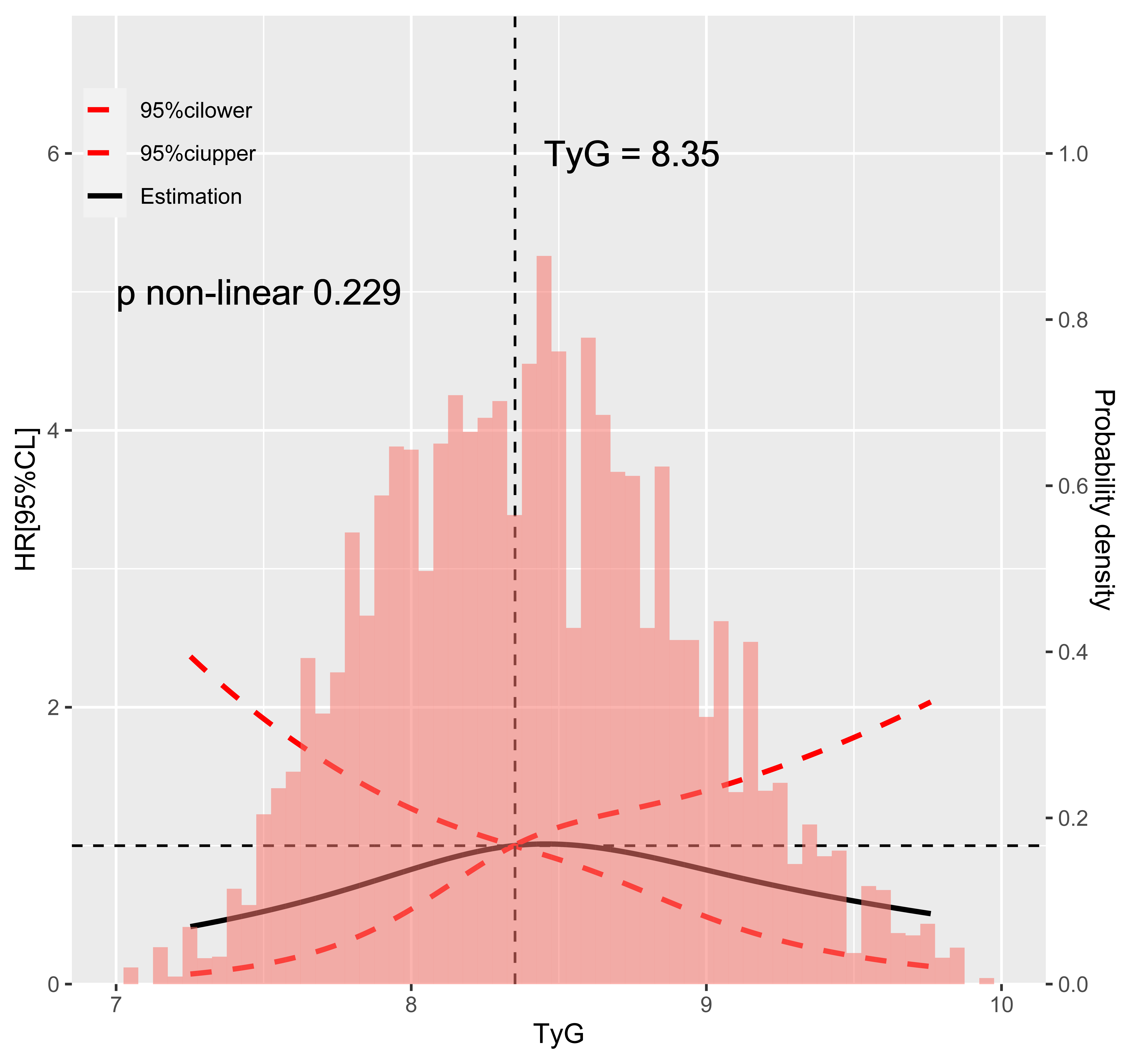
**

The data were analyzed by fitting Cox regression models with weights, utilizing a restricted cubic spline featuring three knots positioned at the 10th, 50th, and 90th percentiles of the baseline TyG index. The model adjusted for age, sex, race, waist circumference, physical activity, current smoking, metabolic syndrome, SBP, DBP, HbA1c, LDL-C, HDL-C, CRP, Hb, and platelet count.

**Figure S3 The association between TyG index and cardiovascular mortality**

**
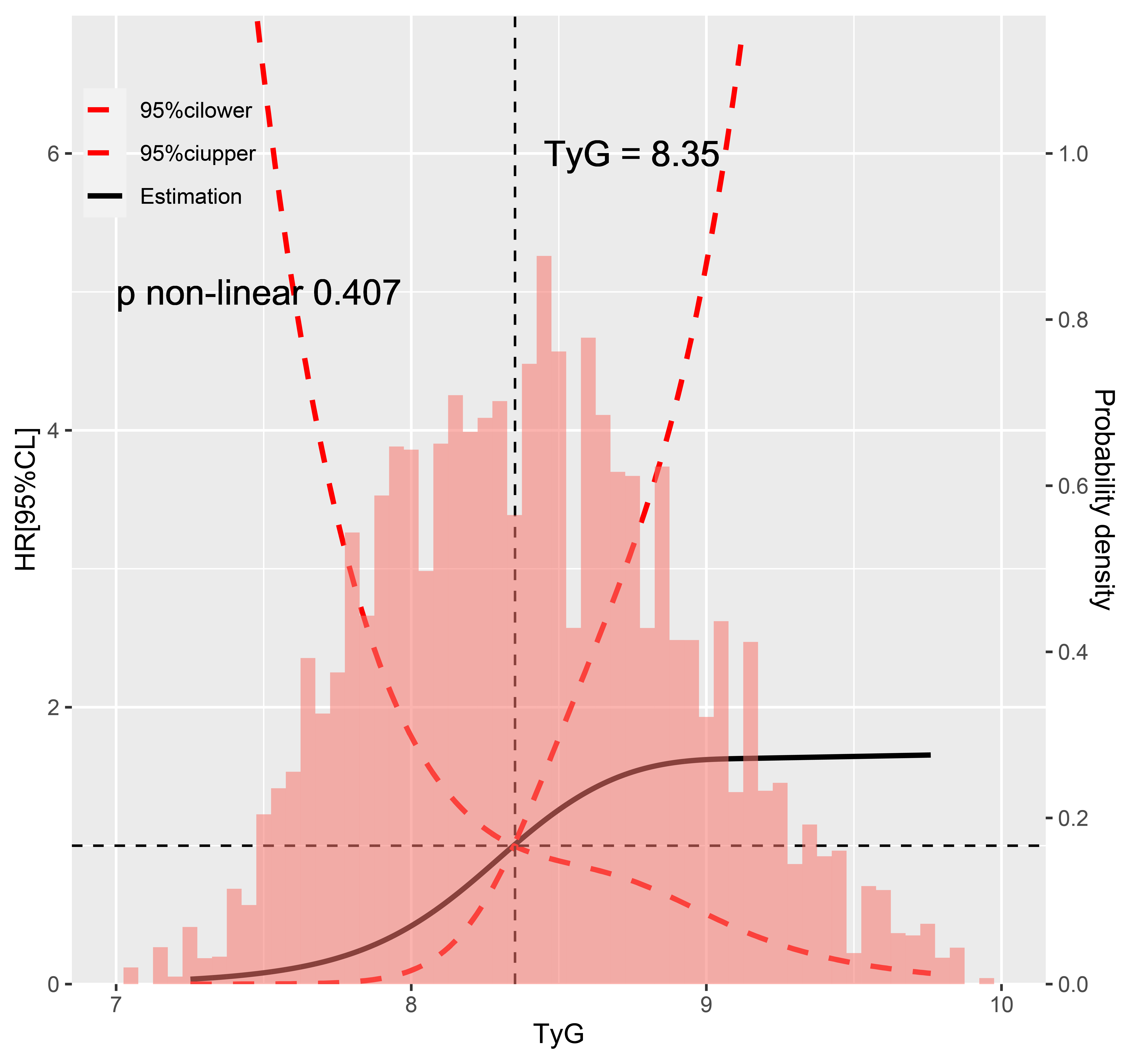
**

The data were analyzed by fitting Cox regression models with weights, utilizing a restricted cubic spline featuring three knots positioned at the 10th, 50th, and 90th percentiles of the baseline TyG index. The model adjusted for age, sex, race, waist circumference, physical activity, current smoking, metabolic syndrome, SBP, DBP, HbA1c, LDL-C, HDL-C, CRP, Hb, and platelet count.

**Figure S4 Sensitivity analysis 1**





The 'jomo' package was employed to create five sets of interpolated data for filling in missing values. Subsequently, when these five interpolation datasets were assessed using restricted cubic splines, their outcomes were observed to align consistently with the results obtained from the dataset after removing missing values. Master dataset is the dataset with removing missing values. The interpolated datasets 1 to 5 were generated using the 'jomo' package.

**Figure S4 Sensitivity analysis 2**





Participants with FPG >7.0 mmol/L or HbA1c >6.5% were categorized as diabetic patients and subsequently excluded. Simultaneously, the 'jomo' package was utilized for data imputation, and a comparative analysis was performed between the imputed data and the dataset where missing values had been directly removed. This analysis employed restrictive cubic spline analysis. Master dataset is the dataset with removing missing values. The interpolated datasets 1 to 5 were generated using the 'jomo' package.
