# Supplementary material for: Association between the triglyceride-glucose index and impaired cardiovascular fitness in non-diabetic young population
Source: Cardiovasc Diabetol. 2024 Jan 20;23:39. doi: 10.1186/s12933-023-02089-8 (PMC10800072; doi:10.1186/s12933-023-02089-8)
Supplement: Supplementary file 2 — Additional file 2: Appendix G. Calculation Of Estimated Vo2 Max. [file 12933_2023_2089_MOESM2_ESM.pdf]

APPENDIX G
CALCULATION OF ESTIMATED VO_2 MAX

APPENDIX G. CALCULATION OF ESTIMATED VO_2 MAX

Because the relation between heart rate and oxygen consumption is linear during exercise, we can estimate maximal oxygen consumption ($V0_2$ max) by measuring the heart rate response to known levels of submaximal work. This relation can be summed up by the equation for a line: y = mx + b, where y is heart rate, m is slope, x is $V0_2$, and b is the intercept. By rearranging these terms, we can derive the following equation:

Estimated VO_2max $= \dfrac{\textbf{PMHR – Intercept}}{\textbf{Slope}}$

Slope $= n\,(\Sigma\, x_i\, y_i)$ slope $– (\Sigma\, x_i)\,(\Sigma\, y_i) / n\, \Sigma\, x_i^{\,2} - (\Sigma x_i)^2$
PMHR = 220 - Age at interview
Intercept $= \overline{Y} - b\,\overline{X}$

$\overline{Y}$ = mean of End of Stage 1 Heart Rate + End of Stage 2 Heart Rate
$\overline{X}$ = mean of End of Stage 1 VO_2 + End of Stage 2 VO_2
b = slope

$$Slope = n(\sum x_i y_i) - (\sum x_i)(\sum y_i) / n\sum x_i^2 - (\sum x_i)^2$$

n = 2
$x_i = x_1, x_2$
x_1 = submax VO_2 at end of Stage 1
x_2 = submax VO_2 at end of Stage 2

$y_i = y_1, y_2$
y_1 = end of Stage 1 HR
y_2 = end of Stage 2 HR

Get end of stage $V0_2$ from Appendix H.

Example: A 40 year old man was assigned Protocol 6. Looking in Appendix G, we see that the corresponding submax $V0_2$ values for these stages are 23.3 and 31.4 ml/kg/min respectively. His heart rate at the end of Stage 1 is 120 and at the end of Stage 2 is 140 beats/min respectively. Plugging these numbers into the formulas above, we then solve our equation.

$$\text{Estimated } V0_2 \text{ max} = \frac{180 – 62.47}{2.469} = 47.6 \text{ ml/kg/min}$$
