# Supplementary material for: Association between the triglyceride-glucose index and impaired cardiovascular fitness in non-diabetic young population
Source: Cardiovasc Diabetol. 2024 Jan 20;23:39. doi: 10.1186/s12933-023-02089-8 (PMC10800072; doi:10.1186/s12933-023-02089-8)
Supplement: Supplementary file 3 — Additional file 3. Appendix I. Reference ranges for vo2 max. [file 12933_2023_2089_MOESM3_ESM.pdf]

## APPENDIX I. REFERENCE RANGES FOR $VO_2$ MAX

**Males ages 12-19**

| | |
|---|---|
| Low | < 42 |
| Moderate | 42 – 51.99 |
| High | ≥ 52 |

**Females ages 12**

| | |
|---|---|
| Low | <37 |
| Moderate | 37-45.99 |
| High | ≥ 46 |

**Females ages 13**

| | |
|---|---|
| Low | <36 |
| Moderate | 36-44.99 |
| High | ≥ 45 |

**Females ages 14**

| | |
|---|---|
| Low | <35 |
| Moderate | 35-43.99 |
| High | ≥ 44 |

**Females ages 15-19**

| | |
|---|---|
| Low | <35 |
| Moderate | 35 – 42.99 |
| High | ≥ 43 |

**Males ages 20-29**

| | |
|---|---|
| Low | ≤ 37.13 |
| Moderate | 37.14-44.22 |
| High | ≥ 44.23 |

**Females ages 20-29**

| | |
|---|---|
| Low | ≤ 30.63 |
| Moderate | 30.64-36.64 |
| High | ≥ 36.64 |

**Males ages 30-39**

| | |
|---|---|
| Low | ≤ 35.35 |
| Moderate | 35.36-42.41 |
| High | ≥ 42.42 |

**Females ages 30-39**

| | |
|---|---|
| Low | ≤ 28.70 |
| Moderate | 28.71-34.59 |
| High | ≥ 34.60 |

**Males ages 40-49**

| | |
|---|---|
| Low | ≤ 33.04 |
| Moderate | 33.05-39.88 |
| High | ≥ 39.89 |

**Females ages 40-49**

| | |
|---|---|
| Low | ≤ 26.54 |
| Moderate | 26.55-32.30 |
| High | ≥ 32.31 |

The level of cardiovascular fitness is categorized based on gender-age specific cut-points of estimated VO2 max. The reference cut-points used for adults 20-49 years are based on data from the Aerobics Center Longitudinal Study (ACLS).[1,2] Low level of CV fitness is defined as an estimated VO2 max below the 20th percentile of the ACLS data of the same gender and age group; moderate fitness is defined as a value between the 20th and 59th percentile, and high fitness level is defined as at or above the 60th percentile. The reference standards used for adolescents and young adults 12-19 years are based on the criteria used in the FITNESSGRAM program.[3,4]

Report of Findings statement: "Compared with other people of your age and sex, your cardiovascular fitness level *is <low, moderate, high>*."

**References**


1. American College of Sports Medicine. (1995). *ACSM's Guidelines for Exercise Testing and Prescription*, 6th edition. Philadelphia, PA: Lippincott Williams and Wilkins Company.

2. Blair, S.N., Kohl, H.W. 3rd, Paffenbarger, R.S. Jr., et al. (1989). *Physical Fitness and All-Cause Mortality. A Prospective Study of Healthy Men and Women.* JAMA, 262(17), 2395-401.

3. Cureton, K.J. and Warren, G.L. (1990). *Criterion-Referenced Standards for Youth Health-Related Fitness Tests: A Tutorial.* Research Quarterly for Exercise and Sport, 61(1), 7-19.

4. Cureton, K.J. and Plowman, S.A. (1999). *Aerobic Capacity Assessments.* In Welk G.J., Morrow, J.R. Jr., and Falls H.B. (Ed.) *Fitnessgram Reference Guide (pp. 66-86).* Dallas, TX: Cooper Institute. Available at: http://dev.cooperinst.org/shopping/PDF%20format/Fitnessgram%20Reference%20Guide.pdf
